# Editorial: Prion and prion-like proteins in neurodegenerative diseases

**DOI:** 10.3389/fnins.2022.1073218

**Published:** 2022-11-03

**Authors:** Mattia Toni, Simona Eleuteri

**Affiliations:** ^1^Department of Biology and Biotechnology “Charles Darwin”, Sapienza University, Rome, Italy; ^2^Beth Israel Deaconess Medical Center, Harvard Medical School, Boston, MA, United States

**Keywords:** prion, prion-like proteins, amyotrophic lateral sclerosis, Huntington's disease, Alzheimer's disease

Prion and prion-like neurodegenerative diseases, as well as Alzheimer's disease (AD), Parkinson's disease (PD), amyotrophic lateral sclerosis (ALS), Huntington's disease (HD) are characterized by peculiar pathological mechanisms. The conditions that favor the conformational modification of prion-like proteins, the mechanisms inducing the formation of misfolded protein and amyloid aggregates, the inability of cellular proteostasis to counteract these events and the propagation of prion-like agents in the nervous system are not yet fully understood. An in-depth knowledge of all these aspects is necessary for understanding the mechanisms underlying these pathologies and for defining pharmacological treatments for the prevention and treatment of these neurodegenerative diseases. This special issue entitled “Prion and prion-like proteins in neurodegenerative diseases” contains reviews and research articles aimed at the description and understanding of these mechanisms.

Prion and prion-like diseases are characterized by the deposition of misfolded proteins or amyloid aggregates as internal inclusion (i.e., PD and ALS) or external plaques (i.e., AD) in the CNS. The core of these insoluble deposits is made up of misfolded proteins that are unique to each disease and share no obvious homology with one another. These pathologies are therefore characterized by the involvement of proteins, with a physiological role still not well known, expressed by the cells in soluble forms that can undergo conformational changes that lead to the accumulation of insoluble aggregates or fibrils. The reactions that lead to the conversion of soluble proteins into aggregates play a key role in the molecular mechanism underlying the disease. The conformational changes in the misfolded proteins can be due to genetic alterations associated to the different pathologies as well as missense mutations that lead to altered tertiary structure, or expansion of specific repeat tracts (i.e., CAG repeat in exon 1 for HD), duplication of the genes that results in the overexpression of the misfolded proteins and their aggregation.

The works of Meisl et al. and Thellung et al. review the experimental approaches and analysis techniques that are used to establish the mechanisms of aggregation. This topic can be investigated through the use of different systems such as test tubes, *in vitro* live cells and living organism. The research carried out so far has highlighted the difficulty of combining the results obtained in the different systems in a coherent picture and translating the findings to human disease. The timescale involved in the various systems differs by many orders of magnitude and these create difficulties in extracting quantitative mechanistic parameters that can be compared across systems. The authors discuss how these approaches must be modified and adapted to be applicable *in vivo* and review the existing works that have successfully applied mechanistic analysis of protein aggregation in living systems.

Four broad classes of processes can be defined in the conversion and aggregation of prion and prion-like proteins: initiation, growth, multiplication, and removal. Knowledge of these processes in detail is necessary as it is believed that one or a few of them are the ones that actually control the behavior of the process. Accurate knowledge of these processes can lead to the identification of targets for pharmacological intervention. The formation of unfolded proteins in the cell is indicative of a failure of cellular proteostasis and fallacious phagocytosis mechanisms. Thellung et al. describe the mechanisms of protein quality control of prion protein and the involvement of proteostasis failure in the onset of prion diseases and other neurodegenerative conditions.

Another mechanism that must be understood is the propagation mechanism of the prion or prion-like agent that can be transmitted from one cell to another. In fact, if the conformational change and the formation of insoluble aggregates were limited to one or a few cells, the onset of the disease would not occur. Consequently, the propagation within the nervous system plays a fundamental role in the development of these pathologies. Prion and prion like diseases are characterized by long asymptomatic periods before the onset of symptoms. It is believed that the asymptomatic period corresponds to the time required for the propagation of the prion like agent.

The endo-lysosomal system has been already associated to the spreading of misfolded proteins and Valappil et al. investigate the interaction of amyloid-β (Aβ) aggregates at the membrane and its implication on the spreading of pathogenic aggregates to identify the crucial molecular event in AD. They revealed a plausible mechanism of propagation of AD pathology *via* intracellular transfer in association to plasma membrane damage, endo-lysosomal accumulation and actin re-modulation that may open up new inference in AD research. Donnelly et al. summarize the evidence supporting the prion like spreading of the mutant form of huntingtin protein, studying its neurotoxic mechanism that involves endocytic and exocytic pathways, tunneling nanotubules as potential target to identify new disease modifying therapies in HD.

Omic approaches such as transcriptomics and proteomics are nowadays powerful tools that can be used to understand the cellular mechanisms induced by the infections of prion or prion like agents. For this reason, the transcriptional profiling of brain tissue with prion disease has been extensively used to identify molecular mechanisms associated with the development of prion disease. Slota et al. have been used the RNAseq to study the transcriptional changes in micro-dissected CA1 and thalamus brain tissues from prion infected mice identifying a transcriptional signature of reactive astrocytes and synaptic dysfunctions, and providing useful information on how different neuronal populations respond to prion infection, helping in understanding the cellular mechanisms involved. It has previously described that the reduction in the proliferation and pro-inflammatory response of microglia slowed prion disease (Gómez-Nicola et al., 2013) and the ablation of microglia accelerates prion disease without altering accumulation of misfolded proteins (Bradford et al., 2022). Here the authors found that the prion infection leads to the astrocytes' activation toward and associated phenotype of chronic neurodegeneration. They investigated the possibility that the modulation of glia response may provide a target for drug discovery in prion and prion-like pathologies.

## Author contributions

MT and SE wrote the first draft of the manuscript. All authors contributed to manuscript revision, read, and approved the submitted version.

## Funding

This work was funded by Sapienza University of Rome (Progetti di Ricerca 2020 RM1201729CB8436F).

## Conflict of interest

The authors declare that the research was conducted in the absence of any commercial or financial relationships that could be construed as a potential conflict of interest.

## Publisher's note

All claims expressed in this article are solely those of the authors and do not necessarily represent those of their affiliated organizations, or those of the publisher, the editors and the reviewers. Any product that may be evaluated in this article, or claim that may be made by its manufacturer, is not guaranteed or endorsed by the publisher.
